# Physical activity as a health resource: a cross-sectional survey applying a salutogenic approach to what older adults consider meaningful in organised physical activity initiatives

**DOI:** 10.1080/21642850.2021.1986400

**Published:** 2021-10-11

**Authors:** Helena Ericson, Mikael Quennerstedt, Susanna Geidne

**Affiliations:** Faculty of Medicine and Health, School of Health Sciences, Örebro University, Örebro, Sweden

**Keywords:** Healthy aging, physical activity, salutogenesis, well-being, exercise

## Abstract

**Objectives:**

Examine health resources that physically active older adults consider meaningful when participating in physical activity initiatives. Health resources are protective factors, including attitudes, knowledge, material factors or social support, that potentially enable people to understand and make sense of their lives or to cope with life stressors.

**Design and main outcome measures:**

A cross-sectional quantitative study was conducted with two questionnaires used to serve as a compiled ‘ageing well’ survey: the Salutogenic Physical Activity Health Resources Questionnaire (SPAHRQ) and the short form of the Sense of Coherence questionnaire, SOC-13.

**Results:**

The study included 372 participants ranging from 60 to 96 years of age (mean age: 74.4 ± 7 years; 60% women). Social relations, positive energy, the habit of exercising and embodied satisfaction were considered important by more than 70% of the participants. Social relations were the most meaningful health resource for both men and women (89%). Women rated positive energy as a significantly more important consequence of their participation in physical activity than men (W 88%, M 72%; *p *= .001). The three health resources that were considered less important were capability in and about physical activity, self-worth and identity as an exercising person. Those who were more physically active considered social relations, self-worth and the habit of exercising to a higher extent. Participants with higher sense of coherence consider the habit of exercising to a greater extent to be important.

**Conclusions:**

Findings that social relations, positive energy, the habit of exercising and embodied satisfaction were considered important by more than 70% of the participants, can contribute to a wider understanding of health resources that older adults consider meaningful in their participation in organised physical activity initiatives.

## Introduction

The number of people aged 60 years and above has doubled since 1980 (WHO, 2015). With the general increase in life expectancy, there is a growing focus on issues of health and illness associated with higher age (Barnett et al., 2012; Beyer, Wolff, Warner, Schüz, & Wurm, 2015). Research reveals that physical activity provides substantial health benefits, both medical and psychological, for older adults (Koster, Stenholm, & Schrack, 2018; Rai, Jongenelis, Jackson, Newton, & Pettigrew, 2019). It has further been emphasised in countless studies that participating in regular physical activity can delay age-associated morbidity and disability, thus improving quality of life and extending years of independent living (Bauman, Merom, Bull, Buchner, & Fiatarone Singh, 2016; Rhodes, Janssen, Bredin, Warburton, & Bauman, 2017; Taylor, 2014). A key challenge is thus to encourage older adults to be physically active in order to reduce the risk of inactivity, loneliness and the impact of age-related morbidity (Goldman et al., 2013; Karppinen, Laakkonen, Strandberg, Huohvanainen, & Pitkala, 2016).

However, our current knowledge on the relation between physical activity and health is predominantly based on an understanding of what causes or prevents illness and premature death, rather than on what maintains and promotes health (Quennerstedt, 2008). In contrast, research in health psychology and health promotion has pointed to the many benefits that physical activity and participation in physical activities can have for health more broadly, as a result of the protective factors that physical activity reinforces (Johnson & Acabchuk, 2018; Nyman et al., 2018; Reis et al., 2016). Less is known, however, about the various individual and sociocultural resources that older adults develop when participating in different initiatives and activities where movement and physical activity are central, resources which in the context of this article are understood as *health resources* (Idan, Eriksson, & Al-Yagon, 2017; McCuaig & Quennerstedt, 2018; Mittelmark et al., 2017). Health resources are protective factors, including attitudes, knowledge, material factors or social support, that potentially enable people to understand and make sense of their lives or to cope with life stressors (Lindström & Eriksson, 2005; Mittelmark et al., 2017). The salutogenic approach can contribute to, and give a new perspective on, older adults’ participation in physical activity.

### Background/rationale

#### Physical activity in later life

Physical activity is often defined as ‘activity that is part of one’s daily life involving bodily movements and the use of skeletal muscles’ (Bherer, Erickson, & Liu-Ambrose, 2013) often with the outcome of energy expenditure (Caspersen, Powell, & Christenson, 1985). According to global recommendations, to maintain their health status, older adults are advised to participate in 150–300 min of activity every week, in bouts of 10 min or more. At least 150 min should be moderate to vigorous aerobic activity (Chodzko-Zajko et al., 2009; WHO, 2010). This is often expressed as 30 min of brisk walking or an equivalent activity five days a week, although 75 min of vigorous activity spread across the week or a combination of moderate and vigorous activity are sometimes suggested. Physical activity to improve strength should also be done at least two days a week (Sparling, Howard, Dunstan, & Owen, 2015). The 150-min target is widely disseminated to health professionals and the public. Evidence suggests that regular engagement in physical activity helps to ‘protect’ against age-related chronic diseases, but also to preserve older adults’ ability to maintain their independence and freedom of choice, thereby enhancing their quality of life (Baier, 2019). Many people, especially in older age groups, find it difficult to achieve this level of the recommended activity (Sparling et al., 2015; Tucker, Welk, & Beyler, 2011), and regardless of the numerous benefits of regular physical activity, older adults represent one of the least active groups in society across the globe (Beauchamp et al., 2018). Against this backdrop, physical activity promotion in this age group has become a high priority within health policies (Beard et al., 2016; WHO, 2015).

#### Health resources and physical activity in later life

This raises questions about how to create the best facilities and conditions for such activities, and randomised controlled trials (Pahor et al., 2014; Paterson & Warburton, 2010) have shown, for example, that resistance training and other physical activities for older adults increase quality of life and sense of coherence (SOC) (Ericson, Skoog, Johansson, & Wåhlin-Larsson, 2018; Kekäläinen, Kokko, Sipilä, & Walker, 2018). Older adults have further reported that they engage in physical activity for reasons of health, independence, pleasure and social connection, and to resist the devaluation of ageing bodies (Hudson, Day, & Oliver, 2015; Phoenix & Orr, 2014). Group-based participatory activities also appear to contribute significantly to healthy ageing (Haslam, Cruwys, & Haslam, 2014; Haslam, Cruwys, Milne, Kan, & Haslam, 2016), and positive interpersonal relations fostered through group-based activities, including physical activities, are described as appealing to older adults and serving to promote and sustain their participation (Capalb, O’Halloran, & Liamputtong, 2014; Costello, McDermott, Patel, & Dare, 2019; Dare, Wilkinson, Marquis, & Donovan, 2018).

Advantages such as socialising while the activity takes place and meeting other older adults have further been shown to offer benefits in relation to health for example as health resource (Ericson, Quennerstedt, Skoog, & Johansson, 2018), and as possibilities to communication and structuring of everyday life (Wichmann, Brand, Gansefort, & Darmann-Finck, 2019). From a salutogenic perspective, physical activity has also been shown to be a meaningful, comprehensible and manageable way for older women to engage in the ongoing process of maintaining their health through the use of various health resources. Health resources upon which the participants drew were social relations, positive energy, self-worth, capability in and about physical activity, the habit of exercising, identity as an exercising person, and embodied satisfaction (Ericson, Quennerstedt, et al., 2018).

#### Salutogenic theory: a theoretical framework

In order to explore what health resources that physically active older adults consider meaningful when participating in different physical activity initiatives, we turn to salutogenic health theory as developed by Aaron Antonovsky (1979) and further adapted for understanding physical activity and health (Ericson, Quennerstedt, et al., 2018; McCuaig & Quennerstedt, 2018; Quennerstedt, 2008). Salutogenesis is an approach within health promotion research that seeks to understand and explain origins of health rather than origins of disease (Antonovsky, 1979, 1987) with a particular focus on the causes of good health (Suominen, Blomberc, Helenius, & Koskenvuo, 1999). Antonovsky (1979, 1987) describes health as a continuum, with ease at one end and dis-ease at the other, and explains that over the course of their lives people move along this continuum between better or worse health. Antonovsky uses the metaphor of a swimmer in a river to describe the relation between individual and socio-cultural aspects of health, with ‘up river’ being development towards better health and ‘down river’ movement towards worse health. He describes people as swimmers in the river of life and argues that we must all learn how to swim and to stay in the right parts of the river if we are to have the slightest chance of remaining healthy. The metaphor thus provides a picture of how people struggle during life against a more or less rapid current, and have more or less swimming ability and resources for keeping their head above water each day.

Key concepts from salutogenic theory that we use include general resistance resources (GRR), or as we prefer, health resources (HR) (McCuaig & Quennerstedt, 2018), and sense of coherence (SOC) (Antonovsky, 1987). Health resources, as McCuaig and Quennerstedt (2018) describe them, are ‘diverse individual and sociocultural factors, including physical, material, cognitive, emotional, attitudinal, relational and sociocultural resources that provide meaningful and coherent life experiences’ (p. 113), which thus help us coping the stressors of our present life situations. Health resources can be located in the river, in the swimmer or in the relation between the swimmer and the river, and can be understood as protective factors including attitudes, knowledge, material factors or social support that potentially enable people to understand and make sense of their lives or to cope with life stressors (Lindström & Eriksson, 2005; Mittelmark et al., 2017).

A well-known concept within salutogenic theory, SOC can be described as a global orientation that helps people to experience life as structured, manageable and meaningful by mobilising the health resources to which they have access (Lindström & Eriksson, 2005). As Lindström and Eriksson (2005) conclude, SOC has to do with how we understand our situation, as well as how we use available resources to manage and make sense of the situations we live in. It consists of three measurable components: comprehensibility (a belief that the challenge is understood), manageability (a belief that resources to cope are available) and meaningfulness (having the motivation to cope). People with higher SOC, according to Antonovsky, are less vulnerable in the presence of stressors or difficult situations, due to their ability to mobilise the most appropriate resources at their disposal (Antonovsky, 1987).

Numerous studies have shown that higher SOC is associated with healthier lifestyle choices such as coping behaviour, optimism, hope and physical activity (Amirkhan & Greaves, 2003; Suominen et al., 1999; Suominen, Helenius, Blomberg, Uutela, & Koskenvuo, 2001; Suraj & Singh, 2011; Wainwright et al., 2007). A systematic review of studies using the SOC questionnaire further showed that SOC can be understood as a health-promoting resource which enhances resilience and promotes self-rated health, quality of life and well-being (Eriksson & Lindström, 2006). In our study, salutogenic theory is used first as a theoretical framing of health resources, secondly as a way to investigate to what extent older adults who are physically active in a group of peers consider these health resources important, and thirdly – using the well-established SOC questionnaire – as a way to explore whether the health resources and participants’ sense of coherence (SOC) are related.

### Objectives

For this reason, a salutogenic perspective on health and physical activity has been adopted in this study (Antonovsky, 1987; Ericson, Quennerstedt, et al., 2018; Eriksson & Lindström, 2006; Quennerstedt, 2008), with the main aim of examining what physically active older adults consider meaningful when participating in different physical activity initiatives. The study will first investigate to what extent certain health resources are considered meaningful, secondly investigate whether there are any differences in demographic background factors and time spent in physical activity that are related to particular health resources, and thirdly explore whether health resources and participants’ sense of coherence (SOC) are related.

## Methods

### Study design

The study is a cross-sectional quantitative study conducted after approval by the Swedish Ethical Review Authority (Dnr 2019-04818). Older adults participating in ongoing, targeted, organised physical activity initiatives were the sample of interest.

### Setting

The survey was distributed in and around a medium-sized city in Sweden. Initiatives were located after searching for as many as possible of the organised physical activities initiatives to produce a representative sample. The recruitment of initiatives was based on a snowball technique (Etikan, Alkassim, & Abubakar, 2016) where diversified social networks mailing lists and individuals in senior associations and sports clubs were applied as starting points. Scheduled visits were made to the organised initiatives to collect data. Contact was always first taken with the head of the organisation arranging the initiative. After approval, further contact was taken with the head-coach of the specific initiative to receive consent to visit the group, provide information about the study, and invite the active older adults to participate. Initiatives included in the sample included such activities as resistance training, water exercise, group gymnastics, tennis, table tennis, outdoor walking, indoor boule, outdoor golf, indoor golf dancing, senior Tabata, sitting gymnastics, wrestling, walking football and bowling. The surveys were handed out in person by the first author after written and oral information was provided to the participants and their informed consent was received. The sample-selection process used a snowballing strategy, with contacts sometimes leading us to other initiatives to approach, and additional visits for data collection. The study took place from December 2019 to early March 2020 when the data collection had to stop due to the Covid-19 outbreak in early spring 2020. All questionnaires were coded and handled confidentially.

### Participants

All participants were active in ongoing, organised physical activity initiatives arranged by different organisations on a voluntary basis. Older adults in different initiatives were invited to participate in the survey, and it was voluntary to participate and almost everyone did. Eligibility criteria for inclusion were being 60 or more years of age, free from cognitive disabilities, and active in a regular, organised physical activity initiative that takes place weekly. Exclusion criteria were initiatives explicitly arranged as rehabilitation or with the intention to treat injury or disease. The participants could fill out the survey on site or request a postage-paid, addressed envelope. It was also possible to ask questions while filling in the questionnaire.

### Variables

The investigated variables in the multivariate logistic regression models were covariates (independent variables) were participants’ age (continuous), gender, education (categorical), time spent in physical activity (continuous), and SOC-score (continuous). The outcome variables (dependent variables) in the logistic models are the health resources (social relations, positive energy, self-worth, capability in and about physical activity, the habit of exercising, identity as an exercising person, and embodied satisfaction). Education was assessed and classified according to the International Standard Classification of Education (UNESCO, 2003) with (1) low education (at most nine years of school education), (2) medium education (secondary school) and (3) high education (high school and university studies). The self-rated time spent in any physical activity per week was compiled and divided by seven to get an average of time spent in physical activity per day. That was made to compare with the recommendations on physical activity.

### Data sources/measurement

Two questionnaires were used in this study to serve as a compiled ‘ageing well’ survey: (i) the Salutogenic Physical Activity Health Resources Questionnaire (SPAHRQ) developed from a previous in-depth study of health resources and physically active older adults (Ericson, Quennerstedt, et al., 2018), and (ii) the short form of the Sense of Coherence questionnaire, SOC-13 (Antonovsky, 1987; Eriksson & Lindström, 2006). SPAHRQ is based on the application of a theory-driven approach (McCuaig & Quennerstedt, 2018) for exploring what health resources participants characterised as important for maintaining physical activity. The part used for this study consisted of seven specific health resources previously identified in an in-depth study (Ericson, Quennerstedt, et al., 2018); (i) social relations, (ii) positive energy, (iii) self-worth, (iv) capability in and about physical activity, (v) the habit of exercising, (vi) identity as an exercising person, and (vii) embodied satisfaction. These seven identified health resources were operationalised as questions in SPAHRQ (see Table 1). The final version of SPAHRQ was tested together with a pilot group of 14 physically active older adults. The pilot group discussed, suggested and developed response alternatives together with the first author. When salutogenic questions are asked, they focus on what enhances health rather than what creates non-health, like the questions formulated in this part of SPAHRQ (Table 1).

**Table 1.** The seven health resources as they are formulated in SPAHRQ.

Health resources	Characteristics	SPAHRQ ‘Which factors in the physical activity you are engaged in make it meaningful?’
Social relations	Enjoying togetherness, having meetings, and people caring	The social aspects, meeting others
Positive energy	Functioning as a ‘recharge’ and providing strength all day	It gives me new energy and a better mood
Self-worth	Having confidence and faith in yourself	It gives me confidence
Capability in and about physical activity	Awareness and know-how regarding exercise. Feeling comfortable	I learn about physical activity and the body
The habit of exercising	Regular routines, generating structure and meaning in life	It is a good habit for me to be physically active
Identity as an exercising person	Being proud of doing exercise, being validated by others	Physical activity gives me an identity as an exercising person
Embodied satisfaction	View of oneself, physical fitness, liking one’s own body	I feel satisfied with my body if I keep up my physical activity

Note: The questions presented here were to be answered in the form of ‘tic in the box’ if you agree.

SOC-13 (Antonovsky, 1987; Eriksson & Lindström, 2006) is a shorter version of the well-established SOC-29 questionnaire, and contains 13 items (SOC-13) (Antonovsky 1987, 1993). It has shown high internal consistency (Cronbach’s alpha = 0.70–0.92) (Eriksson & Lindström, 2006), and includes all three SOC components or subscales where respondents answer the questions on a bipolar, 7-point rating scale. Example items for comprehensibility, manageability, and meaningfulness respectively are: ‘In the past 10 years, your life has been: 1 (full of changes without your knowing what will happen next) to 7 (completely consistent or clear),’ ‘In the past, when you had to do something which depended upon cooperation with others, did you have the feeling that it: 1 (surely would not get done) to 7 (surely would get done)?’ and ‘How often do you feel that there is little meaning in the things you do in your daily life: 1 (very often) to 7 (very rarely or never)?’ The possible range of scores is 13–91, and in a review of 458 studies using the SOC-13 and published during the years 1992–2003, scores ranged from 35 to 77 points (Eriksson & Lindström, 2006). In the present study, more than 80% of the population had a SOC score of 60 or above, which can be considered a high or strong SOC. Cronbach’s alpha was = 0.86.

### Bias

Using the snowball sampling technique helped us in recruiting a substantial sample in line with the objectives of reaching as many participants as possible in a wide range of physical activity initiatives. To avoid potential bias in using a new measurement, the questionnaire was pilot tested on a similar sample of initiatives. However, this study has some sources of bias which are discussed in the limitations section.

### Study size

To obtain a reasonable sample size eligibility criterion for inclusion was set up in advance as described under section participants. No power calculation was done in advance, since the aim was set to include as wide range of initiatives possible. The number of cases in the sample and number of independent variables included in the model were considered and checked by running descriptive statistics as a first step in an assumption check (Pallant, 2020).

### Quantitative variables

The statistical analyses were performed in The Statistical Package for the Social Sciences, version 26.0 (SPSS Inc., Chicago, IL). All data was coded in SPSS 20.0 database. In order to investigate what physically active older adults consider meaningful when participating in different physical activities, demographic variables, health resources and the SOC-score were analysed. A total composite score for SOC-13 was calculated and means and standard deviations for each scale variable were computed.

### Statistical methods

Multivariate associations were studied using logistic regression analysis with the different health resources as outcome variables (dependent variables) adjusted for age, gender, education, physical activity and SOC (independent variables). Odds ratio (OR) with 95% confidence interval (CI) are reported. Demographics were run with the descriptive variables. Data was checked according to the assumptions suggested in Pallant (2020) before running logistic regressions. Participants in the survey may have occasionally missed to answer some questions, and due to this, there may be slight variation in number of respondents in different analyses, *n* is written for each analysis. Missing data was 8% and was excluded listwise in the logistic analysis. The level of statistical significance was set at *p *<* *.05.

## Results

### Participants

The study included 372 participants ranging from 60 to 96 years of age (mean age: 74.4 ± 7 years; 60% women). Following the additional exclusion of any incomplete questionnaires, a total of *n *=* *342 completed all items for the analysis multivariate logistic regression.

### Descriptive data

The vast majority of the participants were born in Sweden (Table 2). Most lived with a partner in small houses or self-owned apartments. Their education levels were almost equally distributed with some predominance on longer University education as many as 93% of the participants met the general criteria of 150 min/week of physical activity by self-rated data (mean 634 ± 464 min/week) and a majority also had a partner who was physically active. The self-rated time spent in physical activity in this sample is extremely high, and includes all activities in everyday life. More than half of the population lived in larger cities, and the rest lived in smaller towns and the countryside; the majority also lived close to exercise and sport facilities, forested areas, natural environments or bike paths.

**Table 2.** Demographic variables tested in the study, *n *= 372.

Demographics	Proportion
Age 60–69 years	20%
Age 70–79 years	60%
Age 80–96 years	20%
Men	40%
Women	60%
Born in Sweden	95%
Education – Primary school	33%
Education – Secondary school	27%
High school or University	40%
Physical activity >150 min/week	93%
House or self-owned apartment	76%
Rent apartment	24%
Living in a city	54%
Living in a smaller town	31%
Living in the countryside	14%

### Outcome data/main results

#### Health resources: to what extent do older adults consider them meaningful

**Figure 1.**
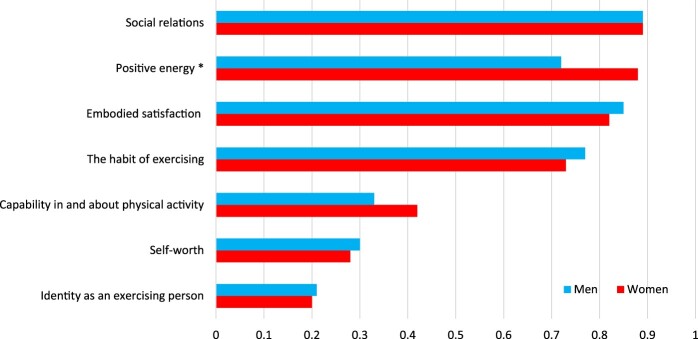
Proportion for each health resource (*n *=* *372).

The health resources social relations, positive energy, the habit of exercising and embodied satisfaction were considered important by more than 70% of both women and men (Figure 1). Women rated positive energy as a significantly more important consequence of their participation in physical activity than men (W 88%, M 72%; *p *=* *.001). The three health resources that were considered less important were capability in and about physical activity, self-worth and identity as an exercising person.

#### Differences in demographic background factors and time spent in physical activity

Time spent being physically active was the covariate that affected which health resource the participants considered most important. Those who were more physically active considered social relations (OR 1.42 (1.09–1.85)), self-worth (OR 1.24 (1.06–1.44)) and the habit of exercising (OR 1.28 (1.07–1.54)) as important to a higher extent than the other participants (Table 3). The outcome variables (dependent variables) in the logistic models (Tables 3 and 4) are the health resources (social relations, positive energy, self-worth, capability in and about physical activity, the habit of exercising, identity as an exercising person, and embodied satisfaction).

**Table 3.** Multiple logistic regression with the different health resources as outcome variables (dependent variables) adjusted for age, gender, education and physical activity (explanatory or independent variables) (*n *= 342).

	Age (cont)	Gender (Reference: Women)	Education (Reference: Primary school)		Physical activity (cont)
		Men	Sec school	University	
Health resources	OR (95% CI)	OR (95% CI)	OR (95% CI)	OR (95% CI)	OR (95% CI)
Social relations	0.94 (0.94–1.05)	1.08 (0.53–2.22)	0.85 (0.33–2.14)	0.70 (0.30–1.60)	**1.42** (**1.09**–**1.85)**
Positive energy	0.98 (0.94–1.03)	**0.42** (**0.23**–**0.76)**	1.15 (0.56–2.36)	1.62 (0.80–3.33)	1.21 (0.98–1.48)
Self-worth	0.98 (0.95–1.01)	1.14 (0.70–1.84)	0.72 (0.34–1.36)	1.24 (0.71–2.15)	**1.24** (**1.06**–**1.44)**
Capability in and about physical activity	1.03 (0.99–1.07)	0.69 (0.44–1.09)	0.87 (0.49–1.55)	1.16 (0.69–1.95)	1.07 (0.93–1.23)
The habit of exercising	0.99 (0.96–1.04)	1.06 (0.63–1.79)	1.83 (0.98–3.42)	**2.75** (**1.51**–**5.02)**	**1.28** (**1.07**–**1.54)**
Identity as an exercising person	1.01 (0.97–1.05)	0.98 (0.58–1.69)	1.79 (0.91–3.51)	1.05 (0.54–2.02)	1.15 (0.97–1.36)
Embodied satisfaction	0.97 (0.93–1.02)	1.35 (0.74–2.46)	0.74 (0.35–1.57)	0.71 (0.36–1.41)	1.31 (1.06–1.61)

Note: *p *<* *.05, written in bold. Nagelkerke *R*-square range between 0.029 and 0.096 for all health resources.

There were no differences between the health resources related to age. Regarding gender, the only difference that emerged was that men regarded positive energy as important to a lesser extent than women (OR 0.42 (0.23–0.76)). The habit of exercising was considered more important for those with a university education than those with primary school education (2.75 (1.51–5.02)).

#### Are the health resources related to participants’ sense of coherence?

Over 80% of the participants in this study had a sense of coherence (SOC) score over 60 (mean SOC 71 ± 10). No differences were found when comparing the highest and lowest 10 deciles of SOC scores, above 84 and under 58 respectively. This was related to which health resources the participants were found to consider important, when bivariate analyses were performed (not presented in tables). When SOC was included in the multiple logistic regression above, none of the above findings changed (Table 4). The only additional significant finding was that participants with higher SOC to a greater extent consider the habit of exercising to be important (1.04 (1.02–1.07)).

**Table 4.** Multiple logistic regression with the different health resources as outcome variables (dependent variables) adjusted for age, gender, education, physical activity and SOC (explanatory or independent variables) (*n *=* *342).

	Age (cont)	Gender (Reference: Women)	Education (Reference: Primary school)		Physical activity (cont)	SOC (cont)
		Men	Sec school	University		
Health resources	OR (95% CI)	OR (95% CI)	OR (95% CI)	OR (95% CI)	OR (95% CI)	OR (95% CI)
Social relations	0.99 (0.94–1.05)	1.03 (0.50–2.15)	0.85 (0.33–2.21)	0.75 (0.32–1.77)	**1.60** (**1.20**–**2.13)**	1.01 (0.97–1.04)
Positive energy	0.98 (0.93–1.02)	**0.41** (**0.22**–**0.75)**	1.01 (0.48–2.14)	1.60 (0.76–3.33)	1.20 (0.96–1.49)	1.02 (0.99–1.05)
Self-worth	0.98 (0.95–1.01)	1.24 (0.75–2.03)	0.64 (0.33–1.26)	1.22 (0.69–2.17)	**1.24** (**1.05**–**1.46)**	1.02 (0.99–1.04)
Capability in and about physical activity	1.02 (0.99–1.06)	0.69 (0.43–1.10)	0.84 (0.46–1.55)	1.28 (0.75–2.18)	1.10 (0.94–1.28)	0.98 (0.96–1.00)
The habit of exercising	0.99 (0.96–1.04)	1.08 (0.62–1.88)	1.67 (0.86–3.24)	**2.50** (**1.33**–**4.70)**	**1.22** (**1.00**–**1.49)**	**1.04** (**1.02**–**1.07)**
Identity as an exercising person	1.00 (0.97–1.04)	1.03 (0.59–1.78)	1.69 (0.83–3.43)	1.09 (0.55–2.14)	1.12 (0.93–1.34)	1.02 (0.99–1.04)
Embodied satisfaction	0.96 (0.92–1.01)	1.31 (0.71–2.41)	0.67 (0.30–1.48)	0.66 (0.32–1.37)	1.35 (1.08–1.68)	0.98 (0.95–1.01)

Note: *p* < .05, written in bold. Nagelkerke *R*-square range between 0.030 and 0.133 for all health resources.

## Discussion

### Key results

Seven health resources were investigated in this study: (i) social relations, (ii) positive energy, (iii) self-worth, (iv) capability in and about physical activity, (v) the habit of exercising, (vi) identity as an exercising person, and (vii) embodied satisfaction. The four most highly rated health resources (see Figure 1) among the participants were social relations, positive energy, embodied satisfaction and the habit of exercising. Social relations, self-worth, and the habit of exercising were more frequent rated for the participants with more time they spent in physical activities in general. Social relations were considered the most meaningful health resource by both men and women (89%). This stresses the importance of social networks for older adults’ health and wellbeing, as Bélanger et al. (2016) and Miller et al. (2019) also argue, regardless of gender. In this context, social relations refer to the social interaction that takes place and is experienced when performing physical activity together with peers. The second most highly rated health resource for the participants was positive energy. Positive energy is to be noted as an added value of being physically active, and was more rated for female participants in our study which was the only significant difference between gender in this study. Positive energy during and after exercise is also highlighted in other studies (Ericson, Skoog, et al., 2018; Kosteli, Williams, & Cumming, 2016; Phoenix & Orr, 2015). The third most highly rated health resource, embodied satisfaction, refers to how participants view themselves and how they appreciate their own bodies. As Phoenix and Orr (2014) argue, pleasure derived from being physically active is something really worth noting also for older adults. The fourth most highly rated health resource was the habit of exercising, which refers to a regular routine that generates structure and meaning in life for the participants. Another finding is that there are no differences in health resources related to age, even though the participants were born during three decades. This result points to equal perceptions of physical activity initiatives, which can imply that they can be constructed similarly among older adults.

Further, the participants with university education rated ‘habit of exercising’ as more important; they also spent significantly more time being physically active and had a stronger sense of coherence. Again, this is a privileged group of older adults who age well and have many possibilities to engage in physical activities.

Regarding the question of whether health resources older adults find meaningful and sense of coherence (SOC) are related, we found that the health resource habit of exercising was significantly related to SOC. We observe similar tendencies with the other health resources, but find no significant relations. This can be interpreted, as also Idan et al. (2017) and Read, Aunola, Feldt, Leinonen, and Ruoppila (2005) emphasise, that socioeconomic status is important when studying health, as habit of exercising was the only health resource significantly related to higher education. Over 80% of the participants had SOC scores above 60, which is often a cut-off point for a high/strong sense of coherence (Amirkhan & Greaves, 2003). Strong SOC and higher education are correlated in other studies as well (Sarit, Rajesh, Eriksson, & Pai, 2020).

A strong SOC can improve one’s readiness to coordinate and take advantage of available health resources (Read et al., 2005; Suominen et al., 1999). According to Antonovsky (1987) SOC strengthens health more than vice versa. Health resources makes certain life experiences possible as conditions in the river of life that reinforce SOC, but access to health resources can also be regarded as a potential consequence of SOC. Antonovsky (1979, p. 19) refers to resources as ‘phenomena that provide one with sets of life experiences characterised by consistency [and] participation in shaping outcomes. Wiesmann and Hannich (2019) show that older adults’ SOC, as a special life strength, can explain resilience in older age and serve as an effective predictor of future positive aging. In other words, and in accordance with our own results, a strong SOC and access to several health resources seem to make life more bearable, which facilitates good adaptation in older age.

This study accordingly focuses on how health is maintained from a salutogenic perspective (Antonovsky, 1996) and contributes knowledge about certain health resources that physically active older adults consider meaningful when participating in different organised physical activity initiatives.

### Limitations

There are several limitations to the present study that should be addressed. It is the first time the SPAHR Questionnaire has been used, which can be considered a limitation when it comes to drawing conclusions. On the other hand, the SPAHR Questionnaire was formed from an in-depth theoretical framework and pilot-tested of asking questions in a certain way to convey salutogenic theory (Antonovsky, 1979; Mittelmark et al., 2017). Another limitation is that the scale items did not have the ability to discriminate, as they only had binary options instead of Likert scales. A third limitation could be that the data are self-rated, and some research shows that participants tend to overrate time spent in physical activity per week, it may not be the case in this age group, since older people’s view of health remains stable when they estimate themselves, according to Galenkamp, Huisman, Braam, and Deeg (2012). The awareness of the positive health outcomes of physical activity might thus be crucial for motivating people to engage in more activity (Alkerwi et al., 2015) and can be considered as bias. We are aware of the mass significance issue, which could make 5% of our tests significant although they were not. However, that did not impact according to the objectives that were aimed to study. Finally, a fourth limitation is that the data collection were stopped due to Covid-19 outbreak in Spring 2020 and a few initiatives planned and booked to visit had to be cancelled.

### Interpretation

Drawing general conclusions from our results that would apply to all older adults would, of course, be hasty, but we believe our study can nevertheless be useful. Our sample reported being physically active to a high extent when we asked them about health, and hence these answers convey a view of how physical activity have promising health outcomes for those who are already active. As a consequence, it extends our knowledge of health in older adults. Our results are to be seen as a contribution to surmounting barriers to older adults’ participation in physical activities. Further work is needed, however, to gain a more complete picture of what might be sustaining factors in a physical activity initiative for older adults. We agree with the implications for practice offered by other scholars, such as Kosteli et al. (2016), who suggest boosting self-efficacy, promoting enjoyable activities, creating similar-age exercising groups, increasing positive reinforcement and improving time management skills as ways to help older adults in the transition to retirement to become more active. Implications for practice and action at the societal and environmental levels highlight the need to battle ageism and create health-promoting and social environments for more older adults than just the privileged sample we studied. Understanding the factors and motives for participation in physical activity initiatives is essential to developing health interventions and strategies (Bauman et al., 2012) for this age group. We can still do more to encourage movement, physical activity in creating these environments and meaningful bodily experiences (Ferreira, Kirk, & Drigo, 2020) which we also found to be health resources for these older adults. In sum, joining groups engaged in initiatives for physical activity may reduce feelings of social isolation and support people through the process of ageing. Physical activity is but one route toward optimising health in older age (Phoenix & Orr, 2017).

This study’s finding that social relations, positive energy, the habit of exercising and embodied satisfaction were considered important by more than 70% of the participants, can contribute to a wider understanding of health resources that older adults consider meaningful in their participation in organised physical activity initiatives. Returning to the river-of-life metaphor, the river can be seen as the health continuum ranging from dis-ease to ease, where we all need different health resources, like those we have explored, in order to swim while keeping our heads above water. In our striving towards better health (up-river), health resources are to be seen as the equipment and circumstances that help us to sustain a good and healthy life. Even though we ‘bring along’ our own health resources in one way or the other, it is also possible for us to access more resources as older adults, in a physical activity context together with others.

### Generalisability

We are aware that because we investigated a highly active segment of the population, it is not possible to generalise to all older adults and the lack of generalisability, which stems from the fact that SPAHRQ is a new set of questions, and the sample is extremely highly active. Another important reason why the results might be somewhat limited in generalisability is that this groups of participants consists of older adults with no cognitive disabilities in a stratum of retirees with time, money, knowledge and previous engagement in physical activity as a way of living and maintaining health. The participants find similar health resources to be important, and as they make up a small sample and are homogeneous regarding what they find meaningful in physical activity, it is not possible to generalise to the older adult population in general. However, Antonovsky’s (1987) point is precisely that we have a lot to learn from those who manage to stay healthy.
